# London Doctors

**Published:** 1874-09

**Authors:** 


### Our Exchanges.

*THE CLINIC—July*, 1874.

London Doctors.—The general practitioner who is a licentiate and not an M.D., has a very different position in England from the doctor and consultant. He has a shop or surgery, dispenses his own medicines, attends to all cases, including midwifery, that come to him, and has very humble notions in regard to the pecuniary value of his services. His charges for medicine include his professional opinion, and his prices range from one to several shillings. The general practitioner in England corresponds to the country doctor in the United States, except that the latter has obtained the title of M.D., from some chartered medical college, or has assumed it in virtue of his individual soverignty. The general practitioner treats ordinary diseases, but in cases of difficulty or danger, especially in important families, the consulting physician is summoned. The spheres of the two classes are widely separated, and the licentiate not being a member or fellow of one of the Royal Colleges, occupies an inferior position socially as well as professionally. It is just as impossible for the consulting physician or surgeon to take cases out of his immediate line of practice, or to dispense medicines, as it is for the general practitioner to set up as consultant without the legal qualification conferred by the colleges. * * *

A large part of the medical practice in England is done by the general practitioners, because their services can be procured at a lower rate, and their time is more available. I am told that in the Queen's household a general practitioner is employed for all the slighter ailments, and the services of her ordinary physician or surgeon, required only in cases of importance. Her Majesty having a keen perception of the main chance, is unwilling to pay guinea fees when shillings will suffice.

The more teasing professional work being done by the general practitioner, the consulting physician has much more leisure and can lead a more genteel life. In the preceding letter I indicated briefly the hours and mode of work of this class. The readers of The Clinic may be interested in further details regarding the figure cut in the world by a London Doctor.

The dining-room—the first floor entered from the hall on the ground floor of a London house—is universally, I believe, used as a reception room for patients, and a smaller room at the end of the hall, as a consultation apartment. All London physicians have a butler, or footman in livery, in attendance. Some of these servants occupy a very confidential position and make engagements for the Doctor. Dr. Gull's man is said to be especially expert in impressing visitors with the great importance of his master, and makes the engagements for the visits days and weeks in advance. The anxious patient may appeal for an immediate interview, for an earlier appointment, but the inexorable attendant says it is impossible, that Sir William cannot be disturbed, that the time named is the earliest period at which he can be seen. Sir William Gull's man is said to be well trained, and it is even hinted that anxious patients are sent laboriously away when Sir Wm. Gull is only taking his morning cigar or whiling away a tedious hour with the last new novel; but this is obviously a slander, originating with envious and less successful doctors. But it is certainly true that the difficulty of seeing Sir William Gull only enhances the desire of his patients to procure his services—an illustration of the social law that men prize most that which is most difficult of attainment.

A successful London doctor observes the same state on his rounds through the city as in his office. Driving a wretched horse in a perishing buggy, or trundling along in an omnibus, or aiding his strength with an occasional pull in a hansom or cab, does not suffice for the needs of a London consultant. He rides in a brougham or clarence carriage, his coachman in livery, his footman on the box. The great man is rolled up to the door of the mansion, he sits majestic until the mercury on the box has dismounted and opened the door of the carriage, and then without haste or impairment of dignified demeanor he steps on the pavement. All this, which may appear unmeaning parade, is perfectly consistent with English notions of the fitness of things, and is in entire harmony with the social usages of London. The younger men ride in a brougham drawn by one horse and driven by a liveried coachman, but unattended by the footman. It was recently made a serious charge in a London Medical Journal, that a Demonstrator of Anatomy in one of the London Medical Schools was seen riding in his brougham with kid gloves on at noon. Such eccentric conduct as this was supposed to be the reason why so many candidates for the recent fellowship examination at the Royal College of Surgeons had been plucked or failed to pass.

The state which must be observed by the London Doctor, cannot be maintained without a certain income. Hence it is that for years his life is one of continual toil and anxiety. The number of people who can pay a guinea fee is not very large relatively even in so rich a city as London, and Englishmen are educated to a keen sense of values. The ordinary consulting office fee is a guinea—equal to six dollars nearly—and for a visit twice that sum. Sir William Gull, Sir William Jenner, and probably a few others, have advanced their fees recently, and now charge two guineas for the office consultation. As the money is paid at the time, and as there are no losses and commissions for collection, it is obvious that a very satisfactory income may be made from a few patients each day. Of course the surgeons get, very often, much larger fees than those named above, which are the rates for ordinary consultation visits. Important operations justify, as is the rule everywhere, very much larger fees—the amount being dependent on the circumstances of the patient and the time and skill involved. As Sir Benjamin Brodie once remarked, the large professional income is not made from large fees, which are in the nature of things exceptional, but from the continual dropping into the purse of small ones. I have been informed by those who are in a position to know, that few London professional incomes reach ten thousand pounds (about fifty thousand dollars.) The incomes of the consultants range from one hundred to five thousand pounds. The latter figure is rarely exceeded, even in London. There is no doubt the great physicians and surgeons of New York make much larger incomes. An illustration of this fact was afforded me very recently in the experience of a gentleman who had consulted Dr. Austin Flint, Sr., in New York, and Sir James Paget, in London. The fee of the former was twenty-five dollars, and of the latter two guineas, or about twelve dollars. There is little doubt that Dr. Flint also sees a much larger number of patients than Sir James Paget. * * *

The mortifications to which new-made baronets are exposed, and the social restraints which so interfere with the intellectual activity of the privileged classes, have rendered many eminent medical men quite indifferent, or indeed, opposed, to entering the titled ranks when the opportunity has been offered them.

